# Global contribution of suicide to maternal mortality: a systematic review protocol

**DOI:** 10.1136/bmjopen-2024-087669

**Published:** 2024-09-16

**Authors:** Emma Simmons, Jenny Gong, Zoe Daskalopoulou, Maria A Quigley, Fiona Alderdice, Siân Harrison, Gracia Fellmeth

**Affiliations:** 1 Medical Sciences Division, University of Oxford, Oxford, UK; 2 National Perinatal Epidemiology Unit, Nuffield Department of Population Health, University of Oxford, Oxford, UK; 3 School of Nursing and Midwifery, Queen's University Belfast, Belfast, UK

**Keywords:** Systematic Review, Maternal medicine, Suicide & self-harm

## Abstract

**Abstract:**

**Introduction:**

Maternal suicide is a significant contributor to maternal mortality with devastating consequences for women, families and society. Maternal mortality reporting systems differ across countries and there is no up-to-date overview of maternal suicide deaths globally. This systematic review aims to synthesise the evidence on maternal suicide. The primary objective is to determine the contribution of suicide towards maternal mortality globally and explore differences between geographical regions. The secondary objectives are to summarise the availability and quality of data globally and to describe how suicide deaths are classified across different countries.

**Methods and analysis:**

This protocol follows the Preferred Reporting Items for Systematic Review and Meta-Analysis Protocols guidelines. Medline, Embase, PsycINFO, Global Health and CINAHL databases and the grey literature were searched with no date or language restrictions. Observational studies, national surveys and reports that present data on maternal deaths due to suicide occurring during pregnancy, intrapartum and in the postpartum period will be included. Screening, data extraction and quality assessment will be conducted independently by two reviewers. Results will be summarised narratively. If sufficient outcome data are available, random-effects meta-analyses will be conducted to determine global pooled estimates of suicide-related maternal mortality rates and the proportion of maternal deaths attributable to suicide.

**Ethics and dissemination:**

Ethical approval is not required for this systematic review. Results will be written up for publication in a peer-reviewed journal and findings will be shared at national and international conferences.

**PROSPERO registration number:**

CRD42023429072.

**STRENGTHS AND LIMITATIONS OF THIS STUDY**

- We will follow rigorous methods and report our methodology and results in accordance with the Preferred Reporting Items for Systematic Reviews and Meta-Analyses guidelines.
- The search strategy is highly comprehensive and was developed with an experienced university librarian.
- We will conduct the search without language or date limitations, allowing us to include non-English publications which are often omitted in systematic reviews.
- We will conduct an extensive grey literature search to identify non-indexed and non-academic publications including maternal mortality databases and surveillance reports.
- A limitation of our review is that we will exclude maternal deaths due to injury, accidents or substance use.

## Introduction

### Rationale

 Maternal suicide, defined as death by suicide occurring during pregnancy or within 1 year of giving birth, is a significant contributor to maternal mortality with devastating consequences for women, families and society. Across a number of high-income countries including the UK and the USA, suicide is one of the leading causes of maternal death.^1 2^ In the UK, the most recent data suggests that between 2019 and 2021 almost 40% of maternal deaths between 6 weeks and 12 months after childbirth were due to suicide.^1^ In lower-resource settings, where rates of all-cause maternal mortality are often higher overall, the contribution of suicide to maternal deaths is less clear. A previous review reported a maternal suicide prevalence of 1% across low-income and middle-income countries (LMICs) with a wide range in prevalence between regions.^3^

**Figure 1:**
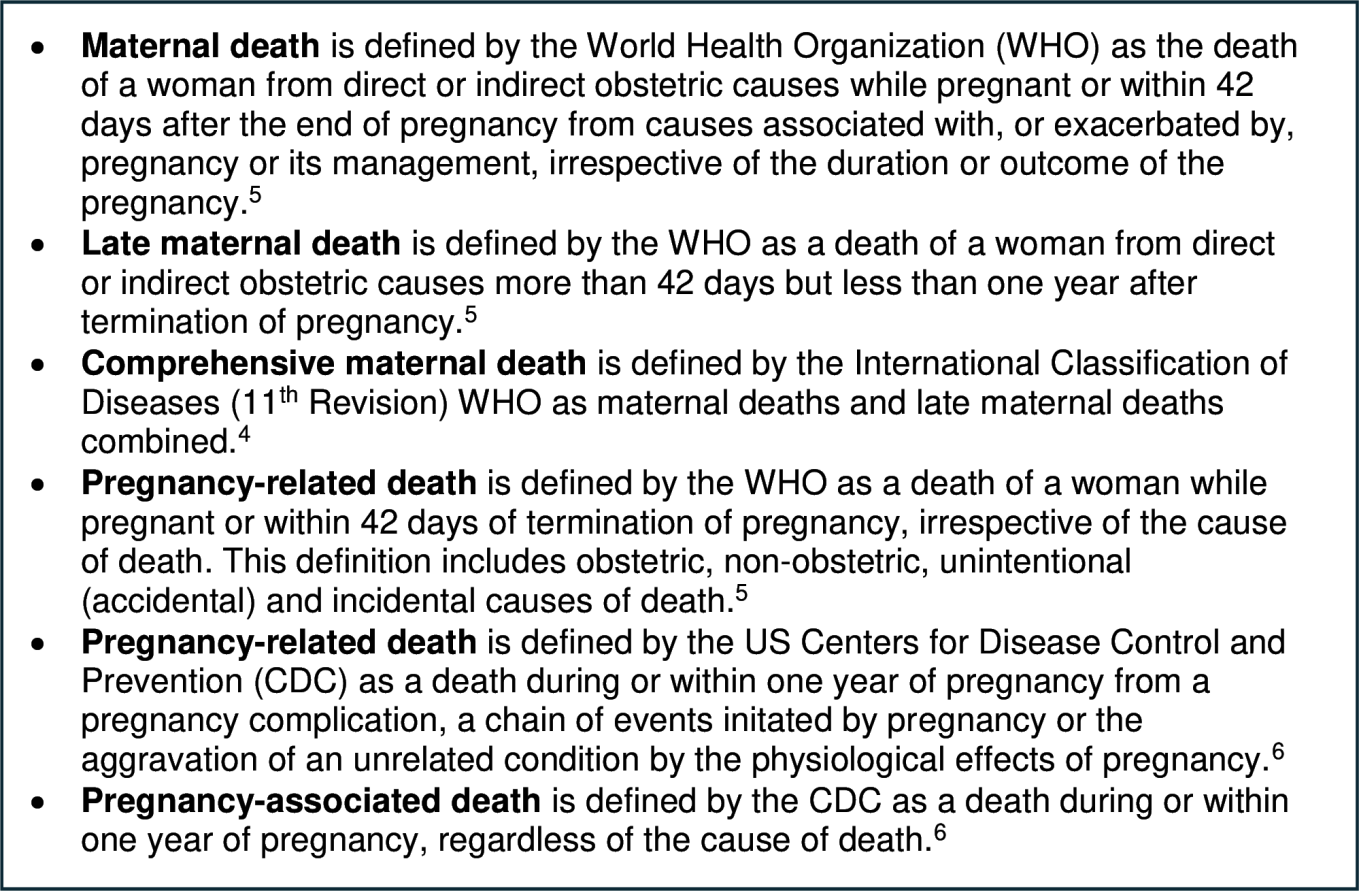
Definitions of maternal death.

Understanding the magnitude of suicide-related maternal mortality is important to improve assessment, identification and reporting of maternal suicide deaths and inform prevention strategies. However, there are a number of challenges in accurately estimating the burden. First, there are a number of different definitions of maternal death (figure 1). The WHO uses the terms maternal death and late maternal death, which are defined in the International Classification of Diseases (11th Revision; ICD-11) and differ from each other only in terms of the time periods they cover.^4 5^ Maternal deaths and late maternal deaths are combined in the ICD-11 under a new grouping of comprehensive maternal deaths.^4^ Others, notably the US Centres for Disease Control and Prevention (CDC), refer to pregnancy-associated and pregnancy-related deaths, which both span the same time period but differ according to the designated cause of death.^6^ Confusingly, the WHO also refers to pregnancy-related death but defines this term differently from the CDC both in terms of the causes included and the time period covered.^5^ In reports and publications presenting maternal death statistics, there is often a lack of clarity around which definition was applied.

Second, there are differences in the denominator used to calculate rates of maternal mortality. The commonly reported maternal mortality ratio (MMR) reports the number of maternal deaths during a given time period per 100 000 live births during the same time period.^5^ The use of live births as a denominator means that deaths occurring during pregnancy and stillbirths are not captured by the MMR. To address this, some countries such as the UK also report the number of maternal deaths per 100 000 maternities, defined as the number of women giving birth, including stillbirth, at or beyond 24 weeks gestation.^1^ When maternities are used as the denominator, the statistic is often referred to as a maternal mortality rate (rather than ratio), but the terminology is inconsistent and in practice the distinction between MMR and maternal mortality rates is often unclear. These differences create challenges when comparing statistics between countries or across different reporting systems.

Third, there is variation in the availability and quality across different regions of the world. High-quality data on maternal suicide require an accurate identification of all maternal deaths, as well as an accurate designation of cause of death. Some countries have systems in place to identify and investigate maternal deaths and their causes at a regional or national level. Such systems include systematic maternal mortality surveillance systems, confidential enquiries and Reproductive Age Mortality Surveys. The assignation of cause of death can include the use of formal death registration certificates and verbal autopsies in which family members are asked to provide information on circumstances around the death. In countries with less robust death reporting systems, it is difficult to determine how accurate maternal death reporting is.

A final challenge is the misclassification of deaths by suicide to other causes such as injury or accidents. This is thought to be common given the stigma associated with suicide and its criminalisation in some societies.^3^ A review of maternal suicide in LMIC found that reclassifying the leading suicide methods from injuries to suicide increased the pooled prevalence of pregnancy-related suicide deaths from 1.00 to 1.68.^3^ Misclassification is compounded by the fact that until the most recent revision of the ICD, maternal suicide did not have a specific code. There has also been a change in the recommended classification of maternal suicides. The ICD-10 categorised maternal deaths due to injuries or mental disorders as indirect maternal deaths.^7^ The WHO subsequently published a derivation of the ICD-10: this ICD-Maternal Mortality (ICD-MM) calls for maternal suicides to be classified as direct maternal deaths.^8^ This discrepancy has led to variations in the reporting of maternal suicide as a direct or indirect cause of death, with some statistics reporting maternal suicide as an incidental cause of death and other statistics excluding suicides from their maternal mortality data altogether.^9^

There are several existing systematic reviews of maternal suicide but these are limited to specific perinatal populations or geographical regions, and there is no up-to-date synthesis of the global evidence for all perinatal women. Lindahl *et al* reviewed prevalence of suicide ideation, attempts and deaths in pregnancy and the postpartum period globally; however, this included studies published up to 2002 and their findings need updating.^10^ Fuhr *et al* assessed the contribution of suicide and injuries to perinatal mortality in LMIC only.^3^ Amiri and Behnezhad assessed rates of suicide ideation, attempts and mortality globally among postpartum women only.^11^ Chin *et al* reviewed prevalence, risk factors, outcomes and interventions for perinatal deaths by suicide but focused on a limited number of studies that were not included in an earlier review by Mangla *et al*.^2 12^ Chin *et al* also restricted their search to studies published in English, and most of the identified studies were conducted in the USA.^2^

Our systematic review expands on previous reviews by including suicides occurring during pregnancy and the postpartum period in all countries globally with no language or date restrictions. Results will enable an estimation of the contribution of suicide to maternal deaths globally and a comparison of the burden across geographical regions. Identifying countries reporting either very high or very low rates will inform further work to determine whether differences in rates reflect true underlying differences or reporting-related or coding-related factors. Our review will also show whether maternal suicide deaths are reported as direct, indirect or incidental maternal deaths, informing a possible need for further clarification of the ICD-11 and ICD-MM classifications. Finally, our review will identify countries for which no data on maternal suicide is available, an important requirement in meeting the United Nations’ Sustainable Development Goal 3.^13^

### Objectives

This systematic review aims to identify and synthesise evidence on maternal suicide deaths globally. The primary objective is to determine the contribution of suicide towards maternal mortality and explore differences between geographical regions. The secondary objectives are to summarise the availability and quality of data globally and to describe how suicide deaths are classified across different countries.

## Methods and analysis

### Registration and protocol adherence

This systematic review protocol is reported in adherence with the Preferred Reporting Items for Systematic Review and Meta-Analysis Protocols guidelines (online supplemental information S1).^14^ The review was registered on PROSPERO (CRD42023429072) on 25 May 2023. If important amendments to the protocol are required during the course of the review, these will be recorded as amendments on PROSPERO and reported in the final publication as a deviation from the protocol. The start date for the review was January 2023 and the planned end date is July 2025.

### Definitions

For the purposes of this review, we define maternal suicide as the death of a woman by suicide at any time during pregnancy, intrapartum or within 1 year of giving birth. The perinatal period is defined as the period of pregnancy and up to 1 year after giving birth.

### Eligibility criteria

#### Population, intervention, comparison and outcome framework

The population of interest is women at any stage of pregnancy or within 1 year of giving birth. The outcome of interest is death by suicide during the perinatal period. There is no intervention or control for this systematic review of global maternal suicide rates.

#### Inclusion and exclusion criteria

We will include any study that reports data on the number of maternal deaths due to suicide along with appropriate denominator data (either total live births or total maternities during the same time period as maternal deaths). Studies reporting the proportion of all maternal deaths that were due to suicide will also be included. We will include cohort, cross-sectional and case–control study designs as well as data from national surveys, databases and reports such as mortality statistics and confidential enquiries. Qualitative studies will be excluded as they are unlikely to contain numerator and denominator data on maternal suicide and maternal death. There will be no minimum threshold applied to the sample size or the number of suicides reported. Studies that do not report data on the denominator to allow calculation of the MMR attributable to suicide or the proportion of deaths that are due to suicide will be excluded. Studies in which suicide deaths cannot be differentiated from deaths from injury or other causes will also be excluded. We will include studies published in any language.

### Patient and public involvement

There was no patient or public involvement in the design of this systematic review.

### Search strategy and data sources

Medline, Embase, PsycINFO, Global Health and CINAHL databases were searched with no date or language restrictions on 5 May 2023. A search strategy using terms relevant to perinatal status, mortality and suicide was developed in collaboration with a university research librarian and adapted for each database. The search strategy used for Medline is shown in table 1. In order to capture relevant evidence not published in the databases above, we conducted an extensive grey literature search. This involved searches of Google and Google Scholar using the relevant terms listed above to capture non-academic articles such as reports and statistical databases. We also searched the websites of the WHO, the UNICEF, the United Nations Population Fund, UN Women, the World Bank and the Global Burden of Disease Study to identify relevant reports. All records identified by the database search and grey literature searches were combined and duplicates were removed using EndNote.^15^ Deduplicated references were imported into Covidence for screening, data extraction and quality assessment.^16^ Prior to submitting the review for publication, the search strategy will be re-run to identify any studies published since the original search.

**Table 1:** Search strategy for Medline

1	Pregnancy/or Pregnant Women/
2	Postnatal Care/or Postpartum Period/
3	1 or 2
4	Mortality/
5	3 and 4
6	maternal mortality/ or maternal death/
7	((pregnan* or maternal or postnatal or post-natal or postpart* or post-part*) and (death? or mortality or fatalit*)).ti.
8	((pregnan* or maternal or postnatal or post-natal or postpart* or post-part*) adj3 (death? or mortality or fatalit*)).ti,ab,kf.
9	5 or 6 or 7 or 8
10	“cause of death”/
11	Death Certificates/
12	exp suicide/
13	“cause of death”.ti,ab,kf.
14	(suicid* or overdos* or self harm or self injury or violen*).ti,ab,kf.
15	10 or 11 or 12 or 13 or 14
16	9 and 15

### Data extraction

Screening of titles and abstracts was performed independently by two reviewers (ES and JG) to exclude studies which did not meet inclusion criteria. Any disagreements were discussed and resolved with a third reviewer (SH and GF). Full texts were retrieved. Any missing full texts were searched for using a university library database, online journal archives, Google and by contacting study authors. Any full texts that could not be retrieved after this search process were excluded. Full texts of the remaining studies were screened independently by two reviewers (ES, JG, SH and GF) and any disagreements were resolved through discussion. Reasons for excluding full texts were recorded. Two reviewers (ES, JG, SH and GF) will independently extract data from included studies using a standardised and piloted data extraction form consisting of the following fields:

- Publication details: lead author, publication year, publication language.
- Setting: country, region, type of setting (eg, hospital or community deaths), dates covered, whether data were collected at national or regional level.
- Participants: perinatal status (pregnant or post partum), definition of maternal death used, age, any sociodemographic characteristics (eg, socioeconomic background, ethnicity, income and parity).
- Methods of assessment: method of identifying maternal deaths, method of ascertaining cause of death, classification of suicide (as indirect, direct or incidental cause of death).
- Outcomes: total maternal deaths, total live births, total maternities, number of suicide deaths, proportion of deaths due to suicide, accuracy measures (eg, SD, p values or 95% CIs), mode of suicide.

If reported data are unclear or insufficient, authors of the study will be contacted to request the additional data required for inclusion in the review. Publications in English, French, German, Greek, Italian, Spanish and Portuguese will be assessed by the study authors. For all other languages, translation support will be sought from wider colleagues.

### Quality assessment and risk of bias

Two reviewers (ES, JG, SH and GF) will independently assess the quality of each included study. We will use criteria adapted from a study by Grollman and Ronsmans^17^ to assess quality and risk of bias. This will include assessment of three domains: quality of the method of death ascertainment; completeness of the cause of death assignation and quality of the method of assigning the cause of death. Each study will be assigned a rating of low, medium or high quality in each of these categories using the attributes outlined by Grollman and Ronsmans.^17^ We have chosen this method as there is a precedent for its use in a similar review, and other risk of bias assessment tools have limited application to studies looking at causes of death. Any disagreements will be discussed with the other review authors (FA and MQ) until a consensus is reached.

### Data synthesis

A narrative synthesis of included studies will be conducted and a table of study characteristics will be compiled to provide an overview of all included studies. Countries for which data on maternal suicide deaths are available will be mapped and colour-coded (red/amber/green) according to the quality of evidence. The proportion of countries classifying maternal suicide deaths as direct, indirect or incidental deaths will also be reported.

Random-effects meta-analyses will be conducted if numerator and denominator data for the same standardised outcome is reported by at least three studies. The primary outcome will be pooled estimates of suicide-related maternal mortality rates. Depending on the data identified and comparability between studies, we will use either live births or maternities as the denominator or conduct a separate pooled estimate for each. Forest plots will be used to present data visually. Statistical heterogeneity between studies will be calculated using the I^2^ statistic. If data allow and sufficient studies are identified, we will carry out subgroup analyses to explore differences in suicide death rates according to perinatal status (pregnancy vs post partum); country income classification (high-income vs LMICs); geographical region (using WHO geographical regions) and study quality. The secondary outcome will be a pooled estimate of the proportion of all maternal deaths caused by suicide, using the number of suicide deaths as the numerator and the total number of maternal deaths as the denominator. If meta-analysis is not possible due to a lack of standardised outcome reporting or due to fewer than three studies presenting the same outcome, results will be reported narratively. The presence of publication bias will be assessed using funnel plots. Meta-analyses will be conducted by using STATA MP version 17.0.^18^

### Ethics and dissemination

No primary data are being collected for this study; therefore, ethical approval is not required. The results of this study will be published in a peer-reviewed publication and presented at conferences. Raw data will be published as online supplemental information S1 in the final publication.

## Discussion

This systematic review and meta-analysis will produce a synthesis of the available evidence on maternal suicide globally. This will allow comparisons to be made between countries on suicide-related maternal mortality rates and the overall contribution of suicide toward maternal mortality. Summarising the availability and quality of data available in different regions of the world will identify priorities for future research. Identifying countries with high or low maternal suicide rates will also inform future studies of risk factors and preventive interventions in different populations.

There are a number of limitations to this study. It is possible that some of the data retrieved will be of poor quality, limiting its reliability and making comparison between areas challenging. The wide variety of methods and definitions used in studies identifying maternal suicide may also make it difficult to draw direct comparisons, and it may not be possible to calculate pooled estimates. By excluding maternal deaths attributed to accidents, injury or substance use, we may be underestimating the true burden of suicide-related maternal mortality. However, given that it is not possible to establish whether these deaths were intentional or non-intentional, we felt it was a safer and more robust approach to exclude these deaths. The lack of data in some regions may limit the power of analyses. There has also been difficulty in acquiring the full texts of several of the included abstracts. Every effort was made to identify these texts, however a significant number of texts had to be excluded. This could impact whether we have captured the full breadth of evidence on this topic.

## supplementary material

10.1136/bmjopen-2024-087669 online supplemental file 1
