# Supplementary material for: Global contribution of suicide to maternal mortality: a systematic review protocol
Source: BMJ Open. 2024 Sep 16;14(9):e087669. doi: 10.1136/bmjopen-2024-087669 (PMC11409235; doi:10.1136/bmjopen-2024-087669)
Supplement: Supplementary file 2 [file bmjopen-2024-087669.reviewer_comments.pdf]

PEER REVIEW HISTORY

BMJ Open publishes all reviews undertaken for accepted manuscripts. Reviewers are asked to complete a checklist review form and are provided with free text boxes to elaborate on their assessment. These free text comments are reproduced below.

ARTICLE DETAILS

**TITLE (PROVISIONAL)**	The global contribution of suicide to maternal mortality: a systematic review protocol
**AUTHORS**	Simmons, Emma; Gong, Jenny; Daskalopoulou, Zoe; Quigley, Maria; Alderdice, Fiona; Harrison, Sian; Fellmeth, Gracia

VERSION 1 - REVIEW

**REVIEWER NAME**	Shigemi, Daisuke
**REVIEWER AFFILIATION**	The University of Tokyo
**REVIEWER CONFLICT OF INTEREST**	None.
**DATE REVIEW RETURNED**	22-Jun-2024

**GENERAL COMMENTS**	Thank you for giving me this opportunity to review this interesting manuscript titled “The global contribution of suicide to maternal mortality: a systematic review protocol”. Overall, the manuscript is well-written, and there is a research significance.<br><br>INTRODUCTION<br>1. It clearly describes the research significance of integrating data on the internationally important topic of maternal suicide, which is in a different situation in terms of definitions and aggregation methods. The significance of analyzing the data without limitation of language or time period is also significant.<br><br>METHODS AND ANALYSIS<br>1. The necessary information is detailed.<br><br>DISCUSSION<br>1. It would require an enormous amount of work to analyze in detail the definition of maternal mortality, aggregation methods, and accuracy of data in each country and region. If it takes a long time to publish a paper, one limitation may be that the results obtained may be out of date.

**REVIEWER NAME**	De Backer, Kaat
**REVIEWER AFFILIATION**	King's College London, Department of Women & Children's Health
**REVIEWER CONFLICT OF INTEREST**	None to declare
**DATE REVIEW RETURNED**	03-Jul-2024

**GENERAL COMMENTS**	Thank you for the opportunity to review this protocol, in a topic that is highly relevant and important. The protocol is clear, with a solid plan for analysing and synthesising the extracted data. The challenges and limitations are appropriately described but the authors have clearly considered how to mitigate these. Once completed, it has the potential to be a highly important contribution to the literature in the field of maternal suicide and mortality. One consideration that was not included in the protocol, is the authors' approach towards deaths from substance overdose, as some of these might be non-accidental and could be classified as suicides. It was not clear if these would be included in the review. This is more a reflection and I have no reservations to accept the protocol in its current form.

**REVIEWER NAME**	Kobylski, Lauren A
**REVIEWER AFFILIATION**	The George Washington University, Psychological & Brain Sciences
**REVIEWER CONFLICT OF INTEREST**	N/A
**DATE REVIEW RETURNED**	09-Jul-2024

**GENERAL COMMENTS**	Thank you for the opportunity to review this manuscript. This review was completed with the assistance of Huynh-Nhu Le, PhD.<br><br>This protocol describes efforts to estimate the global contribution of suicide to maternal mortality, a topic of significant importance. Strengths of the protocol include intentions to include suicides during pregnancy (i.e., not limiting maternal mortality to solely the postpartum period) as well as a maternal suicides globally. The authors will not restrict their search by language or date, addressing major critiques of prior reviews.<br><br>We suggest below some opportunities to further clarify the protocol:<br>1) How will non-English language materials be examined in this review? Will translators or interpreters be involved? (PRISMA-P Item 11)<br>2) Prioritization of outcomes should be delineated. Which outcomes are primary vs. secondary? (PRISMA-P Item 13)<br>3) The authors mention two methods of assessing risk of bias: criteria by Grollman & Ronsmans and criteria by Fuhr et al. It could be more explicit which criteria are ultimately being employed, as the current language seems to suggest the adapted Fuhr et al. criteria will be used, but could also be interpreted as a combination of both versions.<br>4) The authors could clarify how they will know "if sufficient data is available." (PRISMA-P Item 15a)<br>5) Please review the PRISMA-P checklist to ensure that the corresponding page numbers are accurate (e.g., Item 5 is addressed on p. 5-6 but only reported as p. 6, Item 14 is reported as p. 8 but seems to be addressed on p. 7, Item 15d is addressed on p. 7-8 but is only reported as p. 8).

VERSION 1 – AUTHOR RESPONSE

Reviewer 1

Thank you for giving me this opportunity to review this interesting manuscript titled “The global

contribution of suicide to maternal mortality: a systematic review protocol". Overall, the manuscript is well-written, and there is a research significance.
Thank you.

INTRODUCTION: It clearly describes the research significance of integrating data on the internationally important topic of maternal suicide, which is in a different situation in terms of definitions and aggregation methods. The significance of analyzing the data without limitation of language or time period is also significant.
Thank you.

METHODS AND ANALYSIS: The necessary information is detailed.
Thank you.

DISCUSSION: It would require an enormous amount of work to analyze in detail the definition of maternal mortality, aggregation methods, and accuracy of data in each country and region. If it takes a long time to publish a paper, one limitation may be that the results obtained may be out of date.
Thank you for this comment. We agree that the analysis will take a lot of time. To avoid results being out of date, we plan to re-run our search strategy prior to submission. We have added the following statement to the Methods section: *"Prior to submitting the review for publication, the search strategy will be re-run to identify any studies published since the original search."*

Reviewer 2
Thank you for the opportunity to review this protocol, in a topic that is highly relevant and important. The protocol is clear, with a solid plan for analysing and synthesising the extracted data. The challenges and limitations are appropriately described but the authors have clearly considered how to mitigate these. Once completed, it has the potential to be a highly important contribution to the literature in the field of maternal suicide and mortality.
Thank you.

One consideration that was not included in the protocol, is the authors' approach towards deaths from substance overdose, as some of these might be non-accidental and could be classified as suicides. It was not clear if these would be included in the review. This is more a reflection and I have no reservations to accept the protocol in its current form.
Thank you for this helpful comment. When designing our review we decided not to include deaths caused by substance misuse as it would not be possible to establish whether overdoses were intentional or accidental. We acknowledge that this is a limitation and have added it to the 'Strengths and limitations' bullet points as well as the Discussion section: *"By excluding maternal deaths attributed to accidents, injury or substance use, we may be under-estimating the true burden of suicide-related maternal mortality. However, given that it is not possible to establish whether these deaths were intentional or non-intentional, we felt it was a safer and more robust approach to exclude these deaths."*

Reviewer 3
This protocol describes efforts to estimate the global contribution of suicide to maternal mortality, a topic of significant importance. Strengths of the protocol include intentions to include suicides during pregnancy (i.e., not limiting maternal mortality to solely the postpartum period) as well as a maternal suicides globally. The authors will not restrict their search by language or date, addressing major critiques of prior reviews.
Thank you.

We suggest below some opportunities to further clarify the protocol:
1) How will non-English language materials be examined in this review? Will translators or interpreters

be involved? (PRISMA-P Item 11)
As an author team we are able to assess articles in several languages. For other languages, we will seek translation support from our wider network of colleagues. We have added the following sentences to the Methods section: *"Publications in English, French, German, Greek, Italian, Spanish and Portuguese will be assessed by the study authors. For all other languages, translation support will be sought from wider colleagues."*

2) Prioritization of outcomes should be delineated. Which outcomes are primary vs. secondary? (PRISMA-P Item 13)
Thank you. We have now specified our primary and secondary outcomes in the Methods section (under 'Data synthesis'): *"The primary outcome will be pooled estimates of suicide-related maternal mortality rates. […] The secondary outcome will be a pooled estimate of the proportion of all maternal deaths caused by suicide […]."*

3) The authors mention two methods of assessing risk of bias: criteria by Grollman & Ronsmans and criteria by Fuhr et al. It could be more explicit which criteria are ultimately being employed, as the current language seems to suggest the adapted Fuhr et al. criteria will be used, but could also be interpreted as a combination of both versions.
Thank you for raising this. We plan to use the criteria of Grollman & Ronsmans. We have deleted the sentence on Fuhr et al.'s adaptation of these criteria as we agree this was confusing.

4) The authors could clarify how they will know "if sufficient data is available." (PRISMA-P Item 15a)
We have clarified this as follows in the Methods section: *"Random-effects meta-analyses will be conducted if numerator and denominator data on the same standardised outcome is reported by at least three studies."*

5) Please review the PRISMA-P checklist to ensure that the corresponding page numbers are accurate (e.g., Item 5 is addressed on p. 5-6 but only reported as p. 6, Item 14 is reported as p. 8 but seems to be addressed on p. 7, Item 15d is addressed on p. 7-8 but is only reported as p. 8).
Thank you. We have now corrected the page numbers listed in the PRISMA-P checklist.
